# Angiopoietin-1 enhances neutrophil chemotaxis *in vitro* and migration *in vivo* through interaction with CD18 and release of CCL4

**DOI:** 10.1038/s41598-017-02216-y

**Published:** 2017-05-24

**Authors:** Amanda Burnett, Ingrid Gomez, David Davila De Leon, Mark Ariaans, Pavlos Progias, Richard A. Kammerer, Guillermo Velasco, Marie Marron, Paul Hellewell, Victoria Ridger

**Affiliations:** 1 Department of Cardiovascular Science, Faculty of Medicine, Dentistry and Health. University of Sheffield, Sheffield, UK; 2 Department of Biochemistry and Molecular Biology, School of Biology, Complutense University, 28040 Madrid, Spain; 3 Instituto de Investigación Sanitaria del Hospital Clínico San Carlos (IdISSC), Madrid, Spain; 4 Wellcome Trust Centre for Cell-Matrix Research, Faculty of Life Sciences, University of Manchester, Manchester, M13 9PT UK; 5 Laboratory of Biomolecular Research, Division of Biology and Chemistry, Paul Scherrer Institut, CH-5232 Villigen PSI, Switzerland; 6 College of Health and Life Sciences, Brunel University London, Uxbridge, UB8 3PH UK

## Abstract

Angiopoietins are a family of growth factors that are ligands for the tyrosine kinase receptor, Tie2. Angiopoietin 1 (Ang-1) is agonistic for Tie2, plays a key role in blood vessel maturation and stability and has been shown to possess anti-inflammatory properties. However, Tie2 expression has been demonstrated on human neutrophils and the observation that neutrophils migrate in response to Ang-1 *in vitro* has confounded research into its exact role in inflammation as well as its potential use as a therapeutic agent. We used a mouse model of peritoneal neutrophilic inflammation to determine if Ang-1 could stimulate neutrophil migration *in vivo*. Tie2 expression was demonstrated on mouse neutrophils. In addition, recombinant human Ang-1 induced significant chemotaxis of isolated mouse neutrophils in a Tie2- and CD18-dependent manner. Subsequently, co-immunoprecipitation of Ang-1 and CD18 demonstrated their interaction. Intraperitoneal injection of an engineered angiopoietin-1, MAT.Ang-1, induced significant neutrophil migration into the peritoneum and a significant increase in the levels of CCL4 in peritoneal lavage fluid. Depletion of resident peritoneal macrophages prior to, or concomitant injections of an anti-CCL4 antibody with MAT.Ang-1 resulted in a significant reduction in neutrophil recruitment. These data indicate a pro-inflammatory role for Ang-1 with respect to neutrophil recruitment.

## Introduction

The inflammatory process is of great importance in host defence but is also a major contributor to numerous disease processes^1^. During an inflammatory response, neutrophils act as first-responders and are recruited to the injury site. This recruitment is orchestrated by the expression of adhesion molecules on the surface of both the neutrophil and the endothelial cells in addition to the release of chemotactic cytokines that guide the neutrophils to the site of injury^2^.

The angiopoietin family of growth factors are best known for their role in angiogenesis and vascular homeostasis. However, they have also been shown to have effects beyond these conventional roles, specifically in the regulation of vascular inflammation. Ang-1 is a 70 kDa glycoprotein^3^ produced by pericytes^3, 4^. It acts as an agonistic ligand for Tie2 and both are essential for normal vessel development, demonstrating a lethal phenotype in mouse knockout studies^4–6^. Evidence implies that Ang-1 produces a constant basal signal^7^, which maintains the integrity and stability of the endothelium in mature vessels. In addition to this, a number of studies have reported that Ang-1 can inhibit vessel leakage induced by inflammatory agents such as VEGF and mustard oil^8, 9^. Kim *et al*. demonstrated that Ang-1 also inhibits VEGF-induced expression of endothelial cell adhesion molecules ICAM-1, VCAM-1 and E-selectin^10^, which are involved in recruiting leukocytes in inflammatory responses.

As well as its role as a mediator of vascular integrity, a potential alternative role of the angiopoietin/Tie2 system in inflammation has been implicated; expression of Tie2 has been demonstrated on inflammatory cells such as neutrophils^11, 12^, monocytes^13, 14^ and eosinophils^15^. Moreover there have been several studies revealing that the angiopoietins are able to directly activate neutrophils as well as endothelial cells^11, 12, 16^. Sturn *et al*.^12^ demonstrated Tie2-dependent migration of human neutrophils in response to Ang-1 *in vitro* ^12^. Additionally, Lemieux *et al*. (2005) observed that Ang-1 can cause P-selectin translocation to the surface of, and neutrophil adhesion to, endothelial cells through phosphorylation of the Tie2 receptor^11^. However, there is currently no evidence that angiopoietins can act as pro-inflammatory chemoattractants *in vivo*.

Although Ang-1 has been heralded as a potential novel compound for treatment of inflammatory diseases^17, 18^, studies demonstrating the expression of Tie2 on neutrophils and their responsiveness to angiopoietins have highlighted the need for further investigation. We have used a mouse model in order to investigate whether exogenous Ang-1 induces neutrophilic inflammation *in vivo*. We demonstrate that mouse neutrophils express Tie2 and migrate towards recombinant human Ang-1 *in vitro* in a Tie2 and CD18-dependent manner. In addition, the stable MAT.Ang-1 induces neutrophil migration and cytokine release *in vivo*. These novel data demonstrate that Ang-1 can act in a pro-inflammatory manner, not only *in vitro*, but also *in vivo*.

## Results

### Mouse neutrophils express Tie2

**Figure 1:**
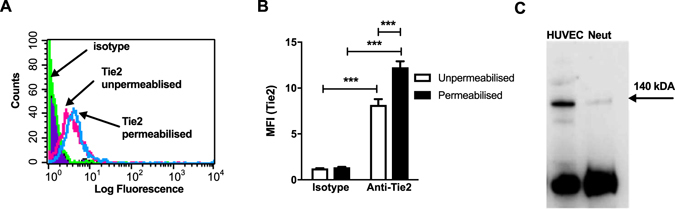
Tie2 expression on mouse peripheral blood neutrophils. (**A**) Ly6G positivity was used to gate on neutrophils using forward and side scatter and Tie2 expression demonstrated as a log shift in fluorescence (FL-2) compared to isotype control. (**B**) For quantification of expression in unpermeabilised and permeabilised neutrophils, the mean fluorescence intensity (MFI) was determined. Results are presented as mean ± SEM (n = 3) and analysed for statistical significance using two-way ANOVA followed by Bonferroni’s post test for multiple comparisons. (**C**) Immunoprecipitation with anti-human Tie2 antibody resolved by SDS-PAGE- and probed with anti-mouse Tie2 antibody. Tie2 protein is detected at approximately 140 kDa. Lane 1: HUVEC lysate (positive control); lane 2: Mouse neutrophil immunoprecipitate.

We first determined whether mouse neutrophils express Tie2. Flow cytometry analysis, using Ly6G positivity to gate on the neutrophil population in lysed mouse whole blood, demonstrated expression of Tie2 on mouse neutrophils (Fig. 1A and B); as previously shown, Tie2 expression was also found on monocytes as previously described^19^ but we were unable to detect its expression on lymphocytes (Data not shown). Permeabilising the cells resulted in an increase in detectable levels of Tie2 (Fig. 1B) suggesting an intracellular source as well as surface expression. The level of Tie2 expression on mouse neutrophils was insufficient for direct detection by immunoblotting. Therefore, immunoprecipitation was used to concentrate the Tie2 protein. Subsequent immunoblot analysis demonstrated that mouse neutrophils express Tie2 protein (Fig. 1C).

### Ang-1 induces neutrophil chemotaxis *in vitro*

**Figure 2:**
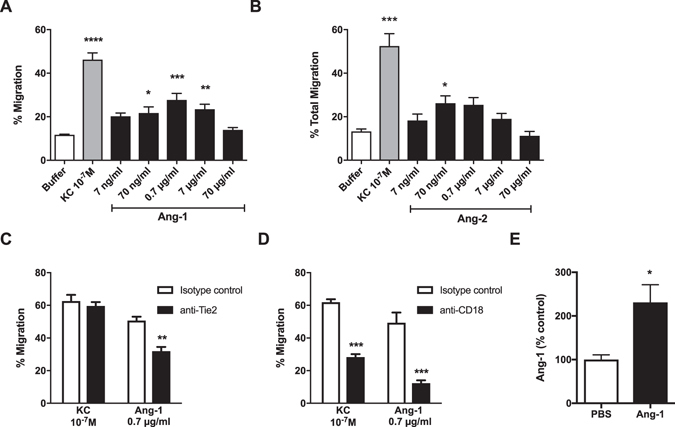
Chemotactic response of mouse neutrophils to rhAng-1 (**A**) and rhAng-2 (**B**). Dose-response effects of rhAng-1 and rhAng-2 on isolated mouse peripheral blood neutrophil chemotaxis. KC (10^−7^ M) was used as a positive control. Results presented as mean ± SEM (n = 4) and analysed for statistical significance using ANOVA followed by Dunnett’s comparing all to buffer control. *P < 0.05 **P < 0.01 ***P < 0.001. The effect of isotype control (rat IgG1), anti-Tie2 (12 µg/ml; 6 µg/10^−6^ cells) (**C**) or anti-CD18 (GAME-46) (**D**) (15 µg/ml; 7.5 µg/10^−6^ cells) on chemotaxis induced by KC (10^−7^ M) or rhAng-1 (0.7 µg/ml). Results presented as mean ± SEM (n = 3–4) and analysed for statistical significance using two-way ANOVA followed by Bonferroni’s test for multiple comparisons. *P < 0.05 **P < 0.01 ***P < 0.001 compared to isotype control. (**E**) Neutrophils were treated with rhAng-1 (0.7 µg/10^6^ neutrophils) or PBS and subjected to extracellular crosslinking. Protein levels of Ang-1 co-immunoprecipitated with CD18 were detected by immunoblotting and quantified using densitometry. Results are expressed as mean ± SEM, n = 3 and analysed for statistical significance using a paired t-test.

Having established that mouse neutrophils express Tie2, we then investigated whether Ang-1 could act as a chemoattractant. We found that Ang-1 significantly (*P* < 0.01) increased mouse neutrophil migration compared to buffer control, in a concentration dependent manner, with a maximal effect observed at 0.7 µg/ml (Fig. 2A). This was similar to the response seen with ﻿Ang-2. Increasing the concentration of Ang-1 resulted in a reduction in chemotaxis. Mouse KC (keratinocyte chemoattractant; 10^−6^ M) was used as a positive control. To determine the role of Tie2 in this response, mouse neutrophils were treated with purified monoclonal rat anti-mouse Tie2 antibody. This resulted in a significant decrease (*P* < 0.01) in neutrophil migration to Ang-1 but not KC (Fig. 2B). However, the response to Ang-1 was only partially attenuated. To further elucidate the mechanism by which Ang-1 induced mouse neutrophil chemotaxis, we investigated the role of the adhesion molecule CD18 (β_2_ integrin) as its role in neutrophil adhesion and migration is well-established^20–22^. Incubation of mouse neutrophils with anti-mouse CD18 (GAME-46 7.5 μg/10^6^ cells) significantly reduced (*P* < 0.001) migration to both Ang-1 and the positive control KC compared to the isotype control (Fig. 2C). Chemoattractants can increase the expression of surface adhesion molecules such as CD18^23^. In order to determine whether Ang-1 acts via increasing surface expression of Tie2 or CD18, neutrophils were incubated with increasing concentrations of Ang-1 and expression determined by flow cytometry. It was found that after 3 h (same exposure as chemotaxis assays) Ang-1 did not induce a significant change in surface CD18 or Tie2 expression (Supplementary Fig. 1). In conclusion, Ang-1 induces neutrophil chemotaxis in a partially Tie2 and CD18 dependent manner that does not involve significant enhancement of surface molecule expression.

### Ang-1 binds to CD18 on neutrophils

Angiopoietins have previously been shown to directly interact with integrins^24, 25^. Therefore, to investigate the mechanism by which Ang-1 could induce chemotaxis in a CD18 dependent manner, we carried out co-immunoprecipitation experiments to determine if Ang-1 was able to directly bind to CD18 on the surface of neutrophils. We found a detectable band of the appropriate size after pull down with anti-CD18 coated beads (Fig. 2D). However, this was at very low levels and required cross-linking in order for the band to be detected (Supplementary Fig. S2). These data suggest that Ang-1 is indeed able to bind directly to surface CD18, although at very low levels or weakly.

### Neutrophils migrate in response to MAT.Ang-1 *in vivo*

**Figure 3:**
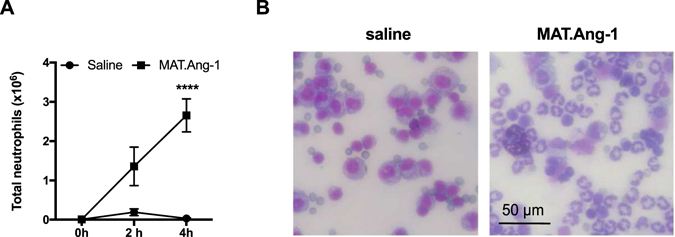
Neutrophil migration in response to MAT.Ang-1 *in vivo*. MAT.Ang-1 (33 µg/mouse) or saline control were injected i.p. into C57/Bl6 mice. After 0–4 h peritoneal lavage was performed and the total neutrophil count was calculated using total leukocyte and differential counts. (**A**) Results are presented as mean ± SEM (n = 3–5 mice) and analysed for statistical significance using two-way ANOVA followed by Bonferroni’s test for multiple comparisons. ***P < 0.001. (**B**) Cytospins were performed using lavage samples taken at 4 h. Hema Gurr® was used to stain and images were taken at ×200 magnification. Scale bar = 50 µm.

MAT.Ang-1 was used in these experiments as it is more stable *in vivo* than recombinant Ang-1^26^, which has been shown to be degraded *in vivo* after 1–2 h. Intraperitoneal injection of MAT.Ang-1 (33 µg/mouse, a dose previously used in mice^27^) induced significant (*P* < 0.001) accumulation of neutrophils in the peritoneal cavity compared to saline controls after 4 h (Fig. 3A). Cytospins produced from the peritoneal lavage fluid of mice injected with saline at 4 h contained few neutrophils in contrast to cytospins from mice injected with MAT.Ang-1 (Fig. 3B). In addition injection of irrelevant human protein (human serum albumin, 33 µg/mouse) did not induce neutrophil migration over 4 h (mean ± SEM = 0.033 ± 0.02 × 10^6^ neutrophils/ml, n = 4).

**Figure 4:**
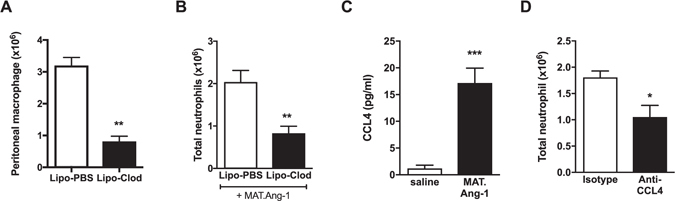
MAT.Ang-1 induced neutrophil migration after depletion of peritoneal macrophages. (**A**) C57/Bl6 mice were administered with 250 µl control or clodronate liposomes i.p. After 48 h, peritoneal lavage was performed and the total monocyte/macrophage count was calculated using total and differential cell counts. (**B**) Liposomes were administered as above 48 h prior to injection of MAT.Ang-1 (33 µg/mouse i.p.). After 4 h, peritoneal lavage was performed and the total neutrophil count was calculated using total and differential cell counts. (**C**) CCL4 levels in peritoneal lavage fluid were measured by cytometric bead array 4 h after MAT.Ang-1 administration (**D**) Rat anti-mouse CCL4 (MAB451) or rat IgG2a (both 50 µg/ml) were administered together with MAT.Ang-1 and neutrophil levels in lavage fluid measured after 4 h. Results are expressed as mean ± SEM (n = 3–5 mice). Statistical significance was analysed using an unpaired t-test. *P < 0.05, **P < 0.01, ***P < 0.001.

From the above experiments we could not determine whether MAT.Ang-1 was acting directly on neutrophils as a chemoattractant, as found in the *in vitro* experiments, or whether there were indirect effects, for example via inducing release of inflammatory mediators from resident peritoneal macrophages. Therefore, macrophages were depleted from the peritoneal cavity through administration of liposome-encapsulated clodronate^28^ resulting in an 85% reduction in the number of macrophage/monocytes measured by peritoneal lavage (Fig. 4A and Supplementary Fig. S3). This effect was specific to tissue macrophages and no effect on circulating leukocyte levels was observed (Supplementary Fig. S3). Macrophage depletion by injection of clodronate liposomes 48 h prior to the administration of MAT.Ang-1 significantly (*P* < 0.05) reduced neutrophil migration compared to that measured in mice injected with control liposomes, although only a partial reduction was observed (Fig. 4B).

### Role of CCL4 (MIP-1β) in MAT.Ang-1-induced neutrophil migration

Investigation into inflammatory mediators present in the lavage fluid collected 4 h after i.p. injection of MAT.Ang-1 compared to saline showed a significant (*P* < 0.05) increase in the level of macrophage inflammatory protein-1β (MIP-1β) known as CCL4 (Fig. 4C). There were no significant differences between the levels of monocyte chemotactic protein-1 (MCP-1), known as CCL2, between saline and MAT.Ang-1 treated mice (PBS: 30.9 ± 0.9; MAT.Ang-1: 33.2 ± 3.5 pg/ml). Levels of granulocyte macrophage colony-stimulating factor (GM-CSF), interleukin-4 (IL-4), interleukin-13 (IL-13), interleukin-17A (IL-17A), keratinocyte chemoattractant (KC), macrophage inflammatory protein-1α (MIP-1 α) or tissue necrosis factorα (TNFα) were below the detection limit for both saline and MAT.Ang-1 injected mice. To determine whether CCL4 release played a role in the neutrophil migration observed after i.p. injection of MAT.Ang-1, a combined injection of MAT.Ang-1 and anti-mouse CCL4 (MAB451) or rat IgG2a isotype control (both given at 50 µg/ml) was administered and peritoneal lavage performed 4 h later. Anti-mouse CCL4 caused a partial, but significant (*P* < 0.05) attenuation of neutrophil migration into the peritoneal cavity compared to isotype control (Fig. 4D). Therefore MAT.Ang-1 induces local release of CCL4 leading to recruitment of neutrophils. In order to determine whether the source of CCL4 was the resident macrophages, CCL4 secretion from isolated peritoneal macrophages was assessed. However, there was no measurable release of CCL4 from peritoneal macrophages after 4 h stimulation with rhAng-1, suggesting that in this *in vitro* model, Ang-1 does not induce CCL4 release from peritoneal macrophages.

## Discussion

In this study we have investigated whether Ang-1 can modulate recruitment of neutrophils *in vivo*. In order to attribute any findings to binding of Ang-1 to Tie2, we have shown expression of Tie2 on mouse neutrophils using immunoprecipitation and flow cytometry although, as with human neutrophils, expression of the receptor was at a very low level^11, 12^. These results add to those of Lemieux *et al*. (2005) who demonstrated Tie2 phosphorylation in human neutrophils by immunoblotting using whole cell lysate with an anti-phospho-specific Tie2 IgG^11^. Interestingly, permeabilisation of neutrophils showed increased Tie2 expression indicating neutrophils have an intracellular store of the receptor, possibly due to receptor internalisation as shown by Bogdanovic *et al*. for endothelial cells^29^.

Tie2 has been shown to be expressed in hematopoietic stem cells^30^. This implies that Tie2 expression is sustained through hematopoietic progenitor cell development into mature neutrophils, which again raises the question of the role of the Tie2/angiopoietin system in the inflammatory response. One possible role is in neutrophil recruitment; Ang-1 has been shown *in vitro* to be chemoattractant for human neutrophils^12, 16^ and andenoviral administration of Ang-1 has been shown to increase neutrophil infiltration in a model of folic acid induced nephrotoxicity in mice^31^. However, another study by Hegeman *et al*. demonstrated rhAng-1 reduced the number of neutrophils present in bronchoalveolar lavage fluid in a mouse model of ventilator-induced lung injury^32^. Here we demonstrate for the first time that mouse neutrophils migrate towards Ang-1 both *in vitro* and *in vivo*. Consistent with other chemoattractants, the migratory response of mouse neutrophils Ang-1 produced a bell shaped curve^33, 34^ with higher concentrations resulting in a reduced migratory response. Our flow cytometry data suggests that this is not a result of a significant reduction in surface expression of either Tie2 or CD18, unlike that described for CXCR1/2 in response to CXCL8^35^.

Tie2 expression on mouse neutrophils is very low and angiopoietins have been shown to bind not only to Tie2 but also to integrins^24, 36, 37^; therefore it was important to show that the chemotaxis observed in mouse neutrophils was specifically mediated via the Tie2 receptor. Treatment of neutrophils with anti-mouse Tie2 antibody significantly reduced Ang-1-induced neutrophil migration. This reduction was specific to chemotaxis induced by rhAng-1 as the response to KC was unaffected, indicating that the mouse neutrophil Tie2 receptor is not involved in the latter response. However, Tie2 inhibition only partially attenuated the chemotactic response to Ang-1 suggesting other mechanisms may also play a role in Ang-1-induced mouse neutrophil migration.

CD18 (β2-integrin) is known to be involved in migration of neutrophils induced by multiple chemoattractants^21, 38^. In addition to the role of Tie2, inhibiting CD18 using a blocking antibody caused migration to be significantly attenuated. This was not due to Ang-1 inducing changes in the surface expression of CD18 on the surface of neutrophils. This is in agreement with published data from Sturn *et al*.^12^ where no significant changes could be detected in L-selectin and CD11b expression using flow cytometry after incubation with angiopoietins^12^. As mentioned above, the angiopoietins are able to interact with integrins directly and we found that this was indeed the case on neutrophils. It is likely that direct interaction of Ang-1 with CD18 plays a role in the migratory response observed but the exact mechanism for this remains to be elucidated. One possibility is that this interaction leads to CD18-mediated signalling that could impact on multiple responses in the neutrophil^39, 40^. Interestingly, the Tie2 receptor has also been shown to interact directly with integrins^36^ and it is possible that clustering of Tie2 with integrins occurs on the cell surface.

Intraperitoneal injection of MAT.Ang-1, at a dose previously shown to reverse LPS-induced inflammation^27^, induced migration of neutrophils into the peritoneal cavity of C57/Bl6 mice. This is the first time Ang-1 has been shown to elicit such pro-inflammatory responses *in vivo*. Depletion of peritoneal macrophages resulted in a partial inhibition of this migration suggesting that MAT.Ang-1 mediates at least some of its effects via stimulation of macrophages. The residual neutrophil migration may have been due to MAT.Ang-1 acting directly on neutrophils, as seen with rhAng-1 *in vitro*. However, as the depletion of macrophages was only approximately 85% it may also be possible that the residual neutrophil migration may have been as a result of MAT.Ang-1 acting upon the remaining 15% of resident cells.

Cytometric bead array analysis of levels of a number of cytokines within the lavage fluid collected 4 hours after injection of MAT.Ang-1 revealed that only levels of CCL4 were significantly higher in the lavage fluid of MAT.Ang-1 injected mice compared to those injected with saline. *In vitro* stimulation of peritoneal macrophages with Ang-1 did not result in a detectable release of CCL4 after 4 hours. Interestingly, Ang-1 has been shown to induce CCL4 expression in neutrophils *in vitro*, but release was found to be delayed, with significantly raised levels of CCL4 only detected after 24 h^41^. However, this may be altered *in vivo*. Blocking CCL4 significantly reduced the amount of neutrophil migration into the peritoneum but the source of CCL4 release remains unknown. However, as with macrophage depletion, there was still some residual neutrophil migration and levels did not return to those observed with saline. This may be due to incomplete inhibition by the antibody, but is more likely an indication that other mechanisms are involved in the response, such as an increase in neutrophil viability as demonstrated by Dumas *et al*.^42^. As both depletion of macrophages and blocking the actions of CCL4 did not completely inhibit neutrophil migration, it is possible that MAT.Ang-1 may act via a direct chemotactic action on neutrophils as seen in the *in vitro* chemotaxis experiments. These data suggest a pro-inflammatory role for Ang-1 with respect to neutrophil migration, which appears contrary to many published studies where administration of Ang-1 *in vivo* attenuates inflammation and acts as a protective mediator^8, 9, 43^. However, this may be due to differences in exogenous Ang-1 compared to endogenous responses; we were unable to detect any Ang-1 in the lavage fluid from thioglycollate induced peritonitis suggesting that Ang-1 does not play a role in neutrophil recruitment in this model of neutrophilic inflammation.

In conclusion, this study demonstrates that Ang-1 has a role as a pro-inflammatory mediator, inducing mouse neutrophil migration both *in vitro* and *in vivo*. *In vitro*, this response was dependent on Tie2 and CD18 but was not due to an alteration in surface receptor expression. Furthermore, our *in vivo* data demonstrates a role for Ang-1 in neutrophil trafficking, potentially through a direct effect on neutrophils, but also through macrophage activation and release of CCL4. This raises the issue of the true role of the angiopoietins in the inflammatory process.

## Materials and Methods

### Animals

Male C57BL/6 (Harlan, Oxford, UK) mice were used. All procedures were approved by the University of Sheffield ethics committee and performed in accordance with the UK Home Office Animals (Scientific Procedures) Act 1986. All cardiac punctures were performed under sodium pentobarbital anesthesia, and all efforts were made to minimize suffering.

### Mouse and human peripheral blood neutrophil isolation

Mouse peripheral blood neutrophils were isolated using a modified version of the negative immunomagnetic separation technique described by Cotter *et al*.^44^. Peripheral blood was incubated with the following monoclonal antibodies: anti-mouse CD2 (1.5 µg/10^6^ lymphocytes), anti-mouse CD5 (2 µg/10^6^ lymphocytes), anti-mouse CD45R (10 µg/10^6^ lymphocytes) (all from Becton Dickinson, Oxford, UK), anti-mouse F4/80 (4 µg/10^6^ monocytes) and anti-mouse CD115 (15 µg/10^6^ monocytes) (both from AbD Serotec, Oxford, UK). Cells were then incubated with goat anti-rat IgG MicroBeads (20 µl/10^7^ cells, Miltenyi Biotec Ltd, Bisley, UK) at 4 °C for 15 min before neutrophils were negatively selected using a magnetic column. Differential counts showed that the neutrophil purity was between 90–98%.

For human studies, all experiments were approved by University of Sheffield Research Ethics Committee (reference SMBRER310) and were performed in accordance with the relevant guidelines and regulations. All subjects gave informed consent. Human peripheral blood neutrophils were isolated using density gradient separation as previously described^45^.

### Mouse peritoneal macrophage isolation

Peritoneal lavage was performed on C57/Bl6 mice and resident macrophages isolated as previously described^46^. CCL4 release was measured after 4 h incubation with recombinant human (rhAng-1, 33 µg/ml; R&D systems, Abingdon, UK) or PBS using a commercial ELISA kit for mouse CCL4 (R&D systems, Abingdon, UK).

### Human umbilical vein cell isolation and culture

Human umbilical vein cells were isolated and cultured as previously described^47^.

### Flow cytometry

Blood was taken from C57/Bl6 mice by cardiac puncture. Red blood cells were lysed using erythrolyse (AbD Serotec, Oxford, UK). The cells were fixed using a kit from Ebiosciences (Hatfield, UK) which has paraformaldehyde based fixation buffer. In addition, samples were permeabilised using buffer containing 0.1% saponin. After treatment, the cells were stained with PE conjugated monoclonal rat anti-mouse Tie2 (clone TEK4, Biolegend, San Diego, CA) or rat IgG1ĸ isotype control. After washing, the samples were analysed using a FACScan flow cytometer (Becton Dickinson, Oxford, UK) and CellQuest Pro software (Becton Dickinson, Oxford, UK). In order to analyse neutrophil specific expression of Tie2, the cells were stained with Ly6G (1A8 - Becton Dickinson, Oxford, UK) allowing the Ly6G^+ve^ cells (neutrophils) to be identified.

Surface expression of Tie2 and CD18 after 3 h incubation with increasing concentrations of rhAng-1 was measured by flow cytometry in a similar manner to that described by Sturn *et al*.^12^ using PE- and FITC-conjugated antibodies against mouse Tie2 or CD18 respectively (Biolegend, San Diego, CA and Becton Dickinson, Oxford, UK respectively).

### Immunoprecipitation and immunoblot analysis

Isolated neutrophils were washed twice with PBS. Cells were lysed (lysis buffer: 50 mM Tris pH 7.5, 50 mM NaCl, 1 mM NaF, 1 mM EGTA, 20% (v/v) Triton-X-100, protease inhibitor), vortexed and centrifuged at 12,000 *g* for 10 min at 4 °C. Protein content of the lysates was measured and equal amounts of protein used in the immunoprecipitation. Protein-G sepharose beads were washed with lysis buffer and 1–2 µg of monoclonal mouse anti-human Tie2 antibody (clone 33; Becton Dickinson Bioscience, Oxford, UK) was added to the beads. Cell lysate was incubated at 4 °C overnight with the antibody treated beads. The beads were pelleted, treated with 10 µl of sample buffer, boiled for 5 min at 95 °C and then resolved by SDS-PAGE. To run the samples, a Nupage™ 4–12% Bis Tris gel 1.0mm x10 well (Sigma-Aldrich, Poole, UK) was used. The membrane was blocked for 1 h using milk powder 5% (w/v) in PBS containing 0.1% (v/v) Tween. The primary antibody (polyclonal rabbit anti-mouse Tie2 antibody; Santa Cruz, Heidelberg, Germany) was added and the membrane was left shaking at 4 °C overnight. Any unbound antibody was removed by washing in PBS containing 0.1% (v/v) Tween three times for 15 min. A horseradish peroxidise (HRP) conjugated secondary antibody was added in PBS containing 0.1% (v/v) Tween for 1 h. The membrane was washed a further three times with PBS containing 0.1% (v/v) Tween. A Supersignal West Dura Extended Duration Substrate (ThermoFisher Scientific, Paisley, UK) was used to provide chemiluminescence for analysis. The blot was analysed on Chemi genius^2^ Bio Imaging System (Syngene, Cambridge, UK).

To investigate the interaction of Ang-1 and CD18, co-immunoprecipitation was performed as previously described^48^ with minimal modifications. Isolated human neutrophils were treated with rhAng-1 (0.7 µg/10^6^ neutrophils) or PBS and the cross linker DTSSP following manufacturer’s instructions (ThermoFisher Scientific, Paisley, UK). Cells were then lysed (40 mM Hepes, 120 mM NaCl, 1 mM EDTA, 10 mM sodium pyrophosphate, 10 mM sodium glycerophosphate, 50 mM sodium fluoride, 0.5 mM sodium orthovanadate, 0.3% CHAPS, 1 mM benzamidine and 0.1 mM PMSF) and the lysates (1–4 mg) precleared by incubating with 5–20 μl of protein G–Sepharose (GE Healthcare Life Sciences, Little Chalfont, UK) conjugated to pre-immune IgG. The lysate extracts and only lysis buffer (negative control) were then incubated with 5–20 μl of protein G–Sepharose covalently conjugated to 5–20 μg of the anti-CD18 IgG (AbD Serotec, Oxford, UK). Immunoprecipitations were carried out overnight at 4 °C on a rotatory wheel. The immunoprecipitates were washed 4 times with lysis buffer, followed by 2 washes with kinase buffer (25 mM Hepes and 50 mM KCl) and resuspended in 30 μl of sample buffer (not containing 2-mercaptoethanol) and filtered through a 0.22-μm Spin-X filter (Sigma-Aldrich, Poole, UK), followed by addition of 2-mercaptoethanol (1% vol/vol). Samples were subjected to electrophoresis and immunoblot analysis with anti-CD18 and anti-angiopoietin-1 antibodies (R&D Systems, Abingdon, UK).

### Neutrophil chemotaxis assay

Migration of mouse neutrophils in response to rhAng-1 (R & D Systems, Abingdon, UK) was performed using a 96-well chemotaxis chamber, which contained a polycarbonate membrane filter with 5 μm pores (NeuroProbe, Gaithersburg, MD) as previously described^45, 49^. A range of concentrations of recombinant human Ang-1 (rhAng-1) diluted in RPMI was placed in the bottom well. The mouse chemoattractant KC (10^−7^ M, Peprotech, London, UK) was used as a positive control. Mouse neutrophils were then added to the upper filter membranes and chemotaxis measured after a 3 h incubation period. The chemotaxis assay was repeated in the presence of saturating concentrations of the monoclonal antibodies anti-Tie2 (12 µg/ml; 6 μg/10^6^ cells; clone TEK4, Biolegend, San Diego, CA,) and anti-CD18 (15 µg/ml; 7.5 μg/10^6^ cells; clone GAME-46, BD Biosciences, Oxford, UK). Isotype controls for each of the antibodies were used at the same concentration. All antibodies were added to the neutrophil suspension dispersed onto the upper filter membrane at the start of the experiment.

### MAT.Ang-1 or thioglycollate induced peritonitis

MAT.Ang-1 was produced as previously described^27, 50^ by substituting the N-terminal with the short coiled-coil domain of chicken matrilin 1 (MAT) fused to the fibrinogen-like domain of native human Ang-1 imparting greater solubility (>95% vs. 60 to 70%) than native Ang-1^26, 50^. To ensure there was no endotoxin contamination, a ProteoSpin endotoxin removal kit was used (Norgen Biotek Corporation, Thorold, Canada). Purified MAT.Ang-1 was used at 33 µg/mouse, a dose previously shown to reverse LPS-induced inflammation in mice^27^. MAT.Ang-1 (33 µg/2 ml) or the equivalent volume of saline or purified low endotoxin human serum albumin (Sigma-Aldrich, Poole, UK) was injected i.p. into C57Bl/6 mice. Alternatively, peritonitis was induced by administration of thioglycollate as previously described^51^. After 0, 2 or 4 h, peritoneal lavage was performed on mice as previously described^51, 52^. The total leukocyte count was calculated using a haemocytometer and the differential count was determined by preparing a cytospin stained with Hema Gurr® Rapid Stain set (VWR, Lutterworth, UK). Cytometric bead array (Becton Dickinson Bioscience, Oxford, UK) or ELISA (mouse Ang-1 PicoKine™ ELISA kit, Boster Biological Technology, Pleasanton, CA) was performed on lavage fluid samples in order to measure any changes in cytokine or Ang-1 content respectively.

### Intraperitoneal macrophage depletion

In order to deplete resident peritoneal macrophages prior to repeating the above peritonitis experiments, C57/Bl6 mice were injected i.p. with 250 µl control or clodronate liposomes (Encapsula Nano Sciences, Nashville, TN) as previously described^53–55^. After 48 h, peritoneal lavage was collected and total leukocyte and differential counts performed as above. Additionally, to analyse any effects of liposome treatment on circulating leukocyte levels, whole blood was taken by cardiac puncture and total leukocyte counts calculated using a haemocytometer. Differential blood counts were determined using a Sysmex hematology analyser (Sysmex Inc, Milton Keynes, UK).

### Statistical Analysis

Results are presented as mean ± standard error (SEM). Statistical analyses were performed using GraphPad Prism version 6 (GraphPad Software, San Diego, CA). One- or two-way analysis of variance followed by Dunnett’s or Bonferroni’s post tests were performed on appropriate data sets. *P* values less than 0.05 were considered significant.

### **Data Availability**﻿

The datasets generated during and/or analysed during the current study are available from the corresponding author on reasonable request.

## Electronic supplementary material

Burnett et al supplementary figures
