# Supplementary material for: Angiopoietin-1 enhances neutrophil chemotaxis in vitro and migration in vivo through interaction with CD18 and release of CCL4
Source: Sci Rep. 2017 May 24;7:2332. doi: 10.1038/s41598-017-02216-y (PMC5443761; doi:10.1038/s41598-017-02216-y)
Supplement: Supplementary file 1 — Burnett et al supplementary figures [file 41598_2017_2216_MOESM1_ESM.pdf]

Angiopoietin-1 enhances neutrophil chemotaxis *in vitro* and migration *in vivo* through interaction with CD18 and release of CCL4

Amanda Burnett, Ingrid Gomez, David Davila De Leon, Mark Ariaans, Pavlos Progias, Richard A. Kammerer, Guillermo Velasco, Marie Marron, Paul Hellewell and Victoria Ridger

Supplementary Information

Supplementary Figure S1

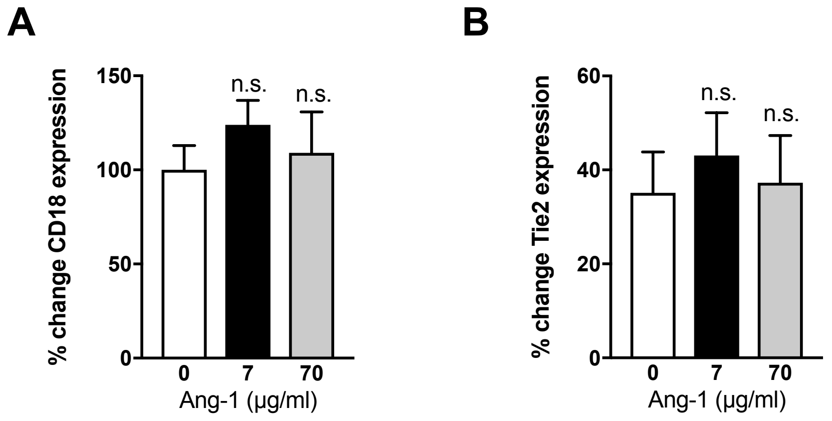

Effect of Ang-1 on mouse neutrophil surface CD18 (A) and Tie2 (B) expression. Neutrophils were treated with increasing concentrations of Ang-1 for 3 h followed by incubation with FITC-onjugated anti-mouse CD18 or PE-conjugated Ti2 and expression analysed by flow cytometry. Data are expressed as mean ± SEM, n=3.

Supplementary Figure S2

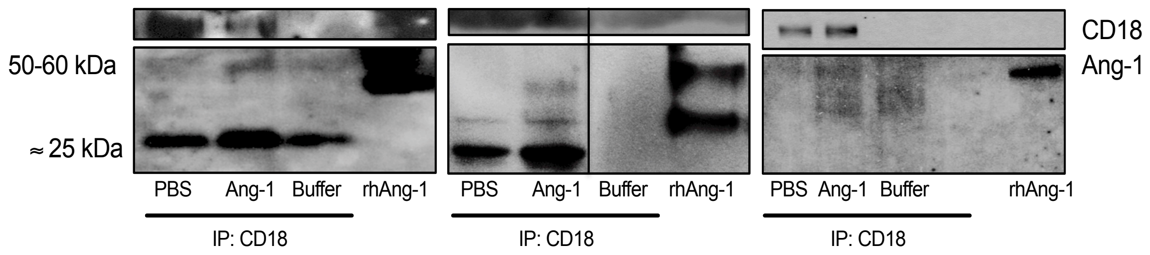

Co-immunoprecipitation of Ang-1 with CD18. Neutrophils were treated with recombinant angiopoietin-1 (0.5 μg/10^6 neutrophils) or PBS and subjected to extracellular crosslinking. Precleared lysates were incubated with anti-CD18 conjugated sepharose beads and immunoprecipitated overnight at 4°C. Protein levels of angiopoietin-1 co-immunoprecipitated with CD18 were detected by western blot. No signal was detected in lysis buffer immunoprecipated as negative control. Blots are from 3 separate experiments.

Supplementary Figure S3

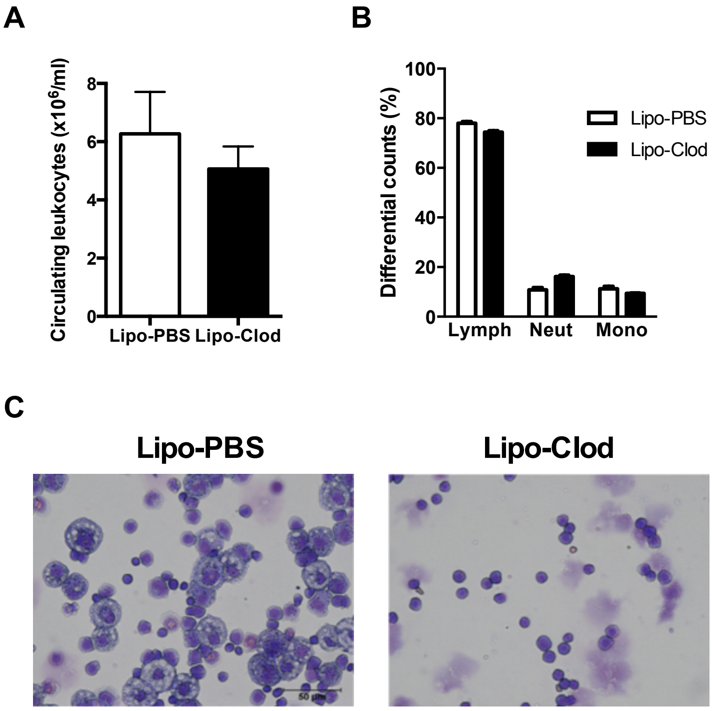

Peritoneal macrophage depletion with clodronate liposomes. Treatment of mice with control or clodronate liposomes had no effect on (A) total circulating leukocyte number or (B) differential counts of lymphocytes, neutrophils and monocytes. (C) Cytospins of peritoneal lavage fluid collected 48 h after liposome injection show a distinct reduction in peritoneal macrophages. Scale bar = 50 μm.
